# Association between maternal lipid profiles and lipid ratios in early to middle pregnancy as well as their dynamic changes and gestational diabetes mellitus

**DOI:** 10.1186/s12884-024-06692-9

**Published:** 2024-07-29

**Authors:** Xingyan Xu, Suping Luo, Jie Lin, Jungu Zhou, Liuyan Zheng, Le Yang, Zhiyu Zhang, Yuting Dong, Mei Ma, Huangyuan Li, Shaowei Lin, Xiaoxu Xie, Jinying Luo, Siying Wu

**Affiliations:** 1 Department of Epidemiology and Health Statistics, School of Public Health, Fujian Medical University, Minhou County, Fuzhou, Fujian China; 2 The Second Attached Hospital of Fujian Medical University, Quanzhou, 362000 China; 3 Department of Fujian Maternity and Child Health Hospital, College of Clinical Medicine for Obstetrics & Gynecology and Pediatrics, Fujian Medical University, Fuzhou, 350001 China; 4 Department of Preventive Medicine, School of Public Health, Fujian Medical University, Minhou County, Fuzhou, 350122 China; 5 Department of Epidemiology and Health Statistics, the Key Laboratory of Environment and Health among Universities and Colleges in Fujian, School of Public Health, Fujian Medical University, Minhou County, Fuzhou, China

**Keywords:** Gestational diabetes mellitus, Lipid profiles, Lipid ratios, Pregnancy, Longitudinal association

## Abstract

**Background:**

Unfavourable lipid and glucose levels may play a crucial role in the pathogenesis of gestational diabetes mellitus (GDM). However, there is a lack of prospective studies on the relationship between lipid profiles, lipid ratios and GDM during pregnancy.

**Aims:**

To prospectively investigate the relationship between lipid profile and lipid ratios in early and mid-pregnancy and their pattern of change from early to mid-pregnancy and the risk of GDM.

**Methods:**

This nested case-control study was based on maternal and child healthcare hospitals from Fujian Province, China. We included pregnant women who delivered in the hospital from January 2021 to June 2023. Lipid profiles (TC, TG, ApoA1, ApoB, HDL-c, LDL-c) and fasting glucose were measured before 14 weeks of gestation and between 20 and 28 weeks of gestation, and lipid ratios (triglyceride glucose index, TG/HDL-c and TC/HDL-c) was constructed. Logistic regression was used to assess the relationship between lipid profile, lipid ratios and GDM.

**Results:**

Of 1586 pregnant women, 741 were diagnosed with GDM. After adjusting for potential confounders, TG, ApoA1, ApoB, LDL-c, triglyceride glucose index, TG/HDL-c, and TC/HDL-c in early pregnancy were positively associated with the risk of GDM (odds ratios [95% CI] for extreme interquartile comparisons were 2.040 (1.468–2.843), 1.506 (1.091–2.082), 1.529 (1.110–2.107), 1.504 (1.086–2.086), 1.952 (1.398–2.731), 2.127 (1.526–2.971), and 2.370 (1.700-3.312), all trend *P* < 0.05). HDL-c was negatively associated with the risk of GDM (0.639: 0.459–0.889, trend *P* all less than 0.05). Similarly, in mid-pregnancy, lower levels of HDL-c, higher levels of triglyceride glucose index, TG/HDL-c ratio, and TC/HDL-c ratio were associated with increased risk of GDM (all trends *P* < 0.05). Stably high levels (both ≥ median for early and mid-pregnancy) of triglyceride glucose index, TG/HDL-c and TC/HDL-c were associated with increased risk of GDM (OR [95% CI]: 2.369 (1.438–3.940), 1.588 (1.077–2.341), 1.921 (1.309–2.829), respectively). The opposite was true for HDL-c, where stable high levels were negatively associated with GDM risk (OR [95% CI]: 0.599 (0.405–0.883)).

**Conclusion:**

Increases in triglyceride glucose index, TG/HDL-c ratio, and TC/HDL-c ratio in early and mid-pregnancy, as well as their stable high levels from early to mid-pregnancy, are associated with a higher risk of GDM. In contrast, increased levels of HDL-c, both in early and mid-pregnancy, and their stable high levels from early to mid-pregnancy were associated with a lower risk of GDM. That highlighted their possible clinical relevance in identifying those at high risk of GDM.

**Supplementary Information:**

The online version contains supplementary material available at 10.1186/s12884-024-06692-9.

## Introduction

GDM is a metabolic disorder caused by insufficient insulin secretion to regulate blood glucose levels during pregnancy, defined as hyper-glycaemia diagnosed for the first time during pregnancy, and is one of the most common pregnancy-related medical complications [1]. The recorded prevalence of GDM varies considerably worldwide, ranging from 1–30% [2], and the global prevalence of GDM is increasing annually as a result of lifestyle changes and updated diagnostic criteria [3]. The prevalence rate in China is about 14.8% [4]. GDM has a variety of negative effects on mothers and their offspring. For mothers, women with GDM have an increased risk of gestational hypertension, pre-eclampsia and caesarean section, as well as an increased long-term risk of type 2 diabetes mellitus (T2DM) and cardiovascular disease [5]. On the other hand, maternal hyper-glycaemia increases the risk of delivering a baby that is larger than gestational age (LGA), shoulder dystocia and birth injuries, which are important determinants of perinatal morbidity and mortality [6]. In addition, offspring of women with GDM have a higher likelihood of developing obesity and glucose metabolism disorders, and cardiovascular disease in childhood or early adulthood [7]. Although some risk factors for GDM, such as maternal obesity, have been identified, its pathophysiology remains elusive.

The relationship between lipid metabolism and GDM has recently attracted attention due to the correlation between carbohydrate and lipid metabolic pathways [8, 9]. Adverse blood lipid status may play a key role in accelerating insulin resistance in various chronic diseases, including obesity, T2DM, and GDM [10]. Clinically, maternal lipid profile, measured by total cholesterol (TC), triglycerides (TG), apolipoprotein A1 (ApoA1), apolipoprotein B (ApoB), high-density lipoprotein cholesterol (HDL-c), and low-density lipoprotein cholesterol (LDL-c), has been associated with insulin-resistant disorders and maintenance of a normal pregnancy [11, 12].

Recently, three lipid ratios, including two triglyceride-related markers (triacylglycerol glucose index and TG/HDL-c ratio) and TC/HDL-c ratio, have been proposed as indicators of insulin resistance [13, 14], which is the main pathophysiology of GDM. Several studies have evaluated the association of these lipid profiles and lipid ratios with the risk of GDM [15, 16]. Another important issue concerns changes in the lipid profile during pregnancy; For example, serum TG levels may increase 2-3-fold during mid-pregnancy. Therefore, the effects of different gestational stages should be considered when exploring the relationship between lipid profiles and blood glucose levels and their associated lipid ratios and GDM. Notably, most previous studies [17, 18] have focused only on lipid levels in early or mid-pregnancy, and many pregnant women do not undergo their first comprehensive prenatal visit until mid-pregnancy, which limits the clinical application of these findings. In addition, there are discrepancies in studies regarding which lipids and lipid ratios are associated with GDM risk [19]. There is a lack of studies on the dynamic changes in lipid profiles and lipid ratios from early to mid-pregnancy associated with subsequent GDM risk.

The primary objective of this study was to prospectively examine the levels of lipid profiles and lipid ratios in early and mid-pregnancy and their dynamic patterns of change from early to mid-pregnancy in association with the risk of GDM.

.

## Methods

### Study population

This study recruited pregnant women from Fujian Provincial Maternity and Child Health Hospital who received prenatal care from early pregnancy and gave birth in the hospital from January 2021 to June 2023. Pregnant women aged 18–40 were recruited when they registered for prenatal care in early pregnancy (gestational weeks < 14 weeks). Those who self-reported severe chronic or infectious diseases or were unable to participate in the survey were excluded.

For the current analysis, we excluded the following subjects: (i) those without GDM diagnostic information (75 g oral glucose tolerance test [OGTT] or diagnosis in medical records); (ii) those who had diabetes or fasting blood glucose ≥ 7.0 mmol/L at baseline; (iii) those without data on early or mid-pregnancy lipid profiles; (iv) those without information on educational level; (v) women with twin pregnancies or stillbirths. Based on this, we designed a nested case-control study. This study has been approved by the Ethics Committee of Fujian Provincial Maternity and Child Health Hospital (Approval No2020KY117). All participants provided written informed consent before enrollment.

### Exposure measurements

This study collected fasting blood samples for biochemical testing in early pregnancy (gestational weeks < 14) and mid-pregnancy (gestational weeks 20–28) at the Clinical Laboratory Department of Fujian Provincial Maternity and Child Health Hospital 1. The main exposures of interest were TC, TG, ApoA1, ApoB, HDL-c, LDL-c, triglyceride glucose index, TG/HDL-c, and TC/HDL-c.

Applications include triacylglycerol glucose index, TG/HDL-c and TC/HDL-c to assess islet resistance.

triacylglycerol glucose index = Ln [TG * FPG/2].

TG/HDL-c ratio is calculated as = TG/HDL-c.

TC/HDL-c ratio is calculated as = TC/HDL-c.

### Diagnosis of GDM

Participants underwent routine screening for GDM with the 75 g OGTT test at 24–28 weeks of gestation. According to the International Association of Diabetes and Pregnancy Study Groups criteria, a pregnant woman was diagnosed with GDM if she met one of the following criteria: (i) fasting glucose ≥ 5.1 mmol/L; (ii) 1-hour glucose ≥ 10.0 mmol/L; or (iii) 2-hour glucose ≥ 8.5 mmol/L [20]. For participants without 75 g OGTT data, GDM was determined by a diagnosis in the medical record.

### Statistical analysis

The characteristics of the study participants were represented by the mean (standard deviation) of continuous variables and the number (percentage) of categorical variables. Differences were evaluated through t-tests and χ-tests between the GDM group and the non-GDM group.

Data were analysed using blood lipid spectrum and lipid ratios in early and mid-pregnancy. The logistic regression model is used to evaluate the correlation between blood lipid spectrum and lipid ratios in early and mid-pregnancy and the risk of GDM. Odds ratios (ORs) and 95% CIs were estimated for each 1 SD increase in lipid profile and lipid ratios, adjusting for relevant covariates. According to common data processing methods in epidemiology, to minimise the effect of extreme values, all participants were divided into 4 groups based on quartiles of lipid profile and lipid ratios, and linear trends were assessed by modelling the median of each quartile of lipid profile and lipid ratios as a continuous variable. We further examined the nonlinear association between blood lipid spectrum and lipid ratios and the risk of GDM using a regression model based on restricted cubic splines. After adjusting for the above covariates, multiple linear regression was used to assess the associations between lipid profile and lipid ratios with the 3 plasma glucose levels (fasting glucose, 1-hour glucose, and 2-hour glucose) at 75 g OGTT.

We analysed the association of changes in lipid profile and lipid ratios with GDM from early to mid-pregnancy. In addition, the effect of the combined categories of lipid profile and lipid ratios (dichotomised into low and high using the respective median as a threshold) in early and mid-pregnancy on the risk of GDM was also investigated. Low levels in early and mid-pregnancy were defined as stable low levels (reference group), low levels in early and high levels in mid-pregnancy as progressive low levels, high levels in early and low levels in mid-pregnancy as decreasing low levels, and high levels in early and mid-pregnancy as stable high levels.

In addition, we investigated the combined effect of pre-pregnancy BMI and early- and mid-pregnancy lipid profiles and lipid ratios (TC, TG, ApoA1, ApoB, HDL-c, LDL-c, triglyceride glucose index, TG/HDL-c and TC/HDL-c) on GDM by adding a product interaction term of pre-pregnancy BMI×lipid profile and lipid ratios (TC, TG, ApoA1, ApoB, HDL-c, LDL-c, triglyceride glucose index, TG/HDL-c and TC/HDL-c) to our model and lipid ratios and their combined effect of parameter changes between the two periods on GDM. Heat maps were constructed to show differences based on combinations of pre-pregnancy BMI and early/mid-pregnancy maternal TC, TG, ApoA1, ApoB, HDL-c, LDL-c, triglyceride glucose index, TG/HDL-c and TC/HDL-c concentrations (red for high prevalence, blue for low prevalence).

Confounders were included if they were previously reported in researches or were found to correlate with the primary outcome. In our analyses, all the birth outcome models were adjusted for potential confounders, including maternal age, pre-pregnancy BMI, gestational age at the time of blood collection in early pregnancy, educational level, parity, marital status, mode of conception, and fertile season. All *P* values were two-sided, and the statistical significance level was 0.05. All analyses were performed using R software (version 4.0.4) with the “rms” and “visreg” packages.

## Results

### Characteristics of study participants

A total of 1586 pregnant women were included in this study and 741 were diagnosed with GDM (Table 1). The mean maternal age was 30.24 ± 4.18 years. Pre-pregnancy BMI (mean) was 21.74 ± 4.51 kg/m2 and blood collection gestational age (mean) was 11.05 ± 1.73 weeks for early pregnancy and 24.22 ± 2.81 weeks for mid-gestation. In our study, none of the women smoke or drink for at least three months before enrolment, thus we did not show the data on smoking and drinking status. Women with GDM were more likely to be older and have a higher pre-pregnancy BMI compared with those without GDM. In early and mid-gestation, pregnant women with GDM had higher levels of TC, triglyceride glucose index, TG/HDL-c, and TC/HDL-c than those in the non-GDM group, whereas HDL-c, on the contrary, had lower levels than those in the non-GDM group. TG, TC, ApoA1, ApoB, HDL-c, LDL-c, triglyceride glucose index, TG/HDL-c, and TC/HDL-c tended to increase from early to mid-pregnancy regardless of GDM status (Figure S1).

**Table 1:** Baseline characteristics of subjects in study

Characteristics	Non-GDM (*n* = 845)	GDM (*n* = 741)	*P* value
Maternal age, year	29.82 ± 4.12	30.65 ± 4.24	< 0.001
Parity			0.185
0	527(62.4)	438(59.1)	
≥ 1	318(37.6)	303(40.9)	
Educational Level			0.321
Middle school or below	68(8.00)	66(8.90)	
High school or technical	98(11.60)	102(13.80)	
Undergraduate or above	679(80.40)	573(77.30)	
Marital status			< 0.001
Married	827(0.98)	700(0.94)	
Other	18(0.02)	41(0.06)	
Mode of conception			0.012
Natural conception	814(96.30)	729(98.40)	
Assisted reproductive conception	31(3.70)	12(1.60)	
Pre-pregnancy BMI, kg/m2	21.23 ± 5.51	22.25 ± 3.50	< 0.001
Fertile season			< 0.001
Spring	448(53.00)	248(33.50)	
Summer	249(29.50)	60(8.10)	
Autumn	15(1.80)	5(0.70)	
Winter	133(15.70)	428(46.70)	
OGTT fasting, mmol/L	4.41 ± 0.28	4.82 ± 0.45	< 0.001
OGTT 1-hour, mmol/L	7.51 ± 1.38	9.93 ± 1.46	< 0.001
OGTT 2-hour, mmol/L	6.41 ± 1.08	8.40 ± 1.43	< 0.001
Early pregnancy			
Gestational age at blood collection, weeks	11.01 ± 1.76	11.09 ± 1.70	0.362
TC (mmol/L)	4.59 ± 0.76	4.67 ± 0.77	0.032
TG (mmol/L)	1.36 ± 0.77	1.52 ± 0.63	< 0.001
ApoA1	1.41 ± 0.24	1.44 ± 0.24	0.003
ApoB	0.73 ± 0.17	0.77 ± 0.17	< 0.001
HDL-c (mmol/L)	1.69 ± 0.30	1.62 ± 0.31	< 0.001
LDL-c (mmol/L)	2.36 ± 0.61	2.43 ± 0.62	0.018
Triglyceride glucose index	1.1 ± 0.37	1.23 ± 0.40	< 0.001
TG/HDL-c ratio	0.84 ± 0.55	0.99 ± 0.55	< 0.001
TC/HDL-c ratio	2.77 ± 0.54	2.95 ± 0.58	< 0.001
Middle pregnancy			
Gestational age at blood collection, weeks	24.30 ± 2.89	24.13 ± 2.72	0.377
TC (mmol/L)	6.19 ± 1.01	6.11 ± 1.10	0.299
TG (mmol/L)	2.42 ± 0.98	2.55 ± 1.13	0.107
ApoA1	1.80 ± 0.27	1.77 ± 0.31	0.228
ApoB	1.04 ± 0.24	1.08 ± 0.24	0.029
HDL-c (mmol/L)	2.00 ± 0.38	1.88 ± 0.37	< 0.001
LDL-c (mmol/L)	3.36 ± 0.82	3.37 ± 0.89	0.891
Triglyceride glucose index	1.64 ± 0.37	1.75 ± 0.4	0.002
TG/HDL-c ratio	1.31 ± 0.83	1.47 ± 1.13	0.031
TC/HDL-c ratio	3.22 ± 1.42	3.32 ± 0.66	0.251

Abbreviations: BMI, body mass index; GDM, gestational diabetes mellitus; TC, total cholesterol; TG, triglyceride; LDL-c, low-density lipoprotein cholesterol; HDL-c, high-density lipoprotein cholesterol; ApoA1, Apolipoprotein AI; ApoB, Apolipoprotein B; OGTT, oral glucose tolerance test

### Association between lipid profiles and lipid ratios and GDM in early pregnancy

**Fig. 1:**
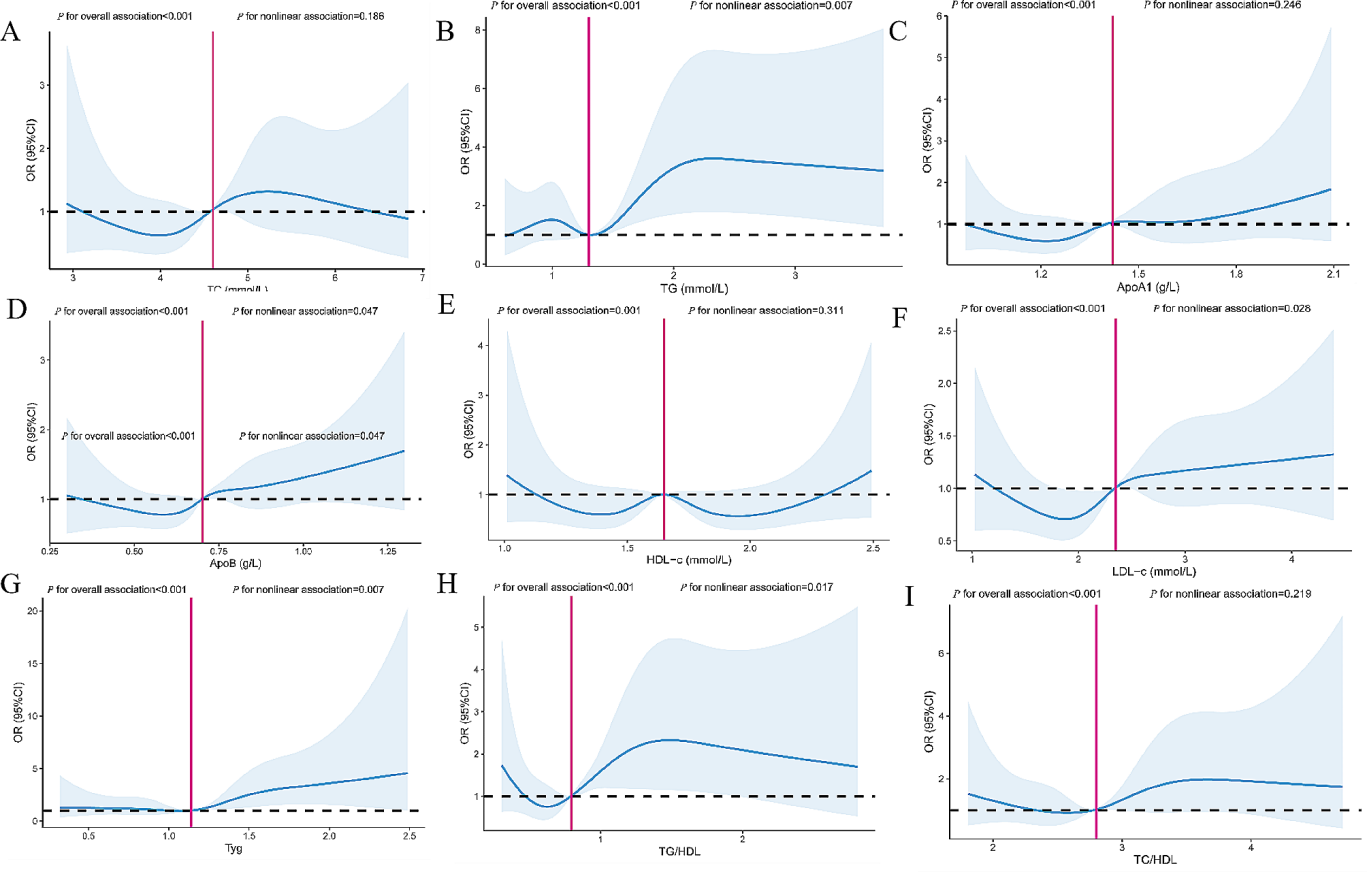
Fig. 1 Restricted cubic splines-based modeling for the associations of maternal lipid profile and lipid ratios in early pregnancy with GDM. The model was adjusted for maternal age, educational level, marital status, pre-pregnancy BMI, parity, mode of conception, gestational age at the time of blood collection in early pregnancy, and fertile season. Panels **A**-I were respectively TC, TG, ApoA1, ApoB, HDL-c, LDL-c, Tyg, TG/HDL and TC/HDL. Abbreviations: BMI, body mass index; GDM, gestational diabetes mellitus; TC, total cholesterol; TG, triglyceride; LDL-c, low-density lipoprotein cholesterol; HDL-c, high-density lipoprotein cholesterol; ApoA1, Apolipoprotein AI; ApoB, Apolipoprotein B

In univariate analyses, per 1-SD increments of TC, TG, ApoA1, ApoB, LDL-c, triglyceride glucose index, TG/HDL-c, and TC/HDL-c in early gestation were positively associated with the risk of GDM, except for per 1-SD increments of HDL-c, which were negatively associated with the risk of GDM (*P* < 0.05, Table 2). After adjusting for maternal age, pre-pregnancy BMI, gestational age at the time of blood collection in early pregnancy, educational level, parity, marital status, mode of conception and fertile season, significant associations between lipid profiles and lipid ratios and GDM remained (all *P* < 0.05). Compared with women in the lowest quartile, the OR (95% CIs) for GDM in women in the highest quartiles of TG, ApoA1, ApoB, HDL-c, LDL-c, triglyceride glucose index, TG/HDL-c, and TC/HDL-c were 2.040(1.468–2.843), 1.506(1.091–2.082), 1.529(1.110–2.107), 0.639(0.459–0.889), 1.504(1.086–2.086), 1.952(1.398–2.731), 2.127(1.526–2.971), and 2.370(1.700-3.312) (P-trend ≤ 0.05, Table 2, Model 2). In the restricted cubic spline-based model, only the associations between TG, APOB, LDL-c, triglyceride glucose index, TG/HDL-c, and GDM were nonlinear (nonlinear *P* < 0.05; Fig. 1), whereas evidence of nonlinear associations for other lipid parameters and indicators of islet resistance was lacking (nonlinear *P* ≥ 0.05, Fig. 1).

**Table 2:** ORs (95% CIs) for GDM according to quartiles of maternal lipid profile and lipid ratios in early pregnancy

Maternal lipid profile and lipid ratios	OR (95%CI) for GDM				*P* for trend*	Per 1-SD increment
	Quartile 1	Quartile 2	Quartile 3	Quartile 4		OR (95%CI)
Early pregnancy						
TC (mmol/L)	3.80 [< 4.11]	4.36 [4.11-<4.57]	4.81 [4.57-<5.09]	5.48 [≥ 5.09]		
Model 1	Reference	0.985 (0.742–1.308)	1.356 (1.023-1.800)	1.275 (0.961–1.693)	0.021	1.155 (1.013–1.318)
Model 2	Reference	0.964 (0.700-1.325)	1.278 (0.930–1.757)	1.332 (0.968–1.834)	0.031	1.176 (1.014–1.366)
TG (mmol/L)	0.90 [< 1.04]	1.17 [1.04-<1.30]	1.46 [1.30-<1.66]	2.00 [≥ 1.66]		
Model 1	Reference	1.059 (0.797–1.407)	1.425 (1.071-1.900)	2.373 (1.783–3.168)	< 0.001	1.509 (1.263–1.820)
Model 2	Reference	1.078 (0.784–1.483)	1.186 (0.860–1.644)	2.040 (1.468–2.843)	< 0.001	1.291 (1.090–1.560)
ApoA1 (g/L)	1.17 [< 1.26]	1.34 [1.26-<1.40]	1.48 [1.40-<1.55]	1.69 [≥ 1.55]		
Model 1	Reference	0.867 (0.654–1.150)	1.179 (0.891–1.562)	1.460 (1.104–1.934)	0.002	1.885 (1.241–2.888)
Model 2	Reference	0.921 (0.670–1.265)	1.136 (0.825–1.565)	1.506 (1.091–2.082)	0.012	1.877 (1.179–3.015)
ApoB (g/L)	0.60 [< 0.60]	0.70 [0.60-<0.70]	0.80 [0.70-<0.80]	0.90 [≥ 0.80]		
Model 1	Reference	1.153 (0.875–1.521)	1.516 (1.139–2.021)	1.581 (1.198–2.091)	< 0.001	3.032 (1.679–5.519)
Model 2	Reference	1.136 (0.834–1.548)	1.428 (1.033–1.976)	1.529 (1.110–2.107)	0.006	2.636 (1.334–5.243)
HDL-c (mmol/L)	1.31 [< 1.45]	1.55 [1.45-<1.64]	1.74 [1.64-<1.86]	2.02 [≥ 1.86]		
Model 1	Reference	0.784 (0.592–1.037)	0.673 (0.509–0.891)	0.545 (0.409–0.725)	< 0.001	0.470 (0.337–0.652)
Model 2	Reference	0.713 (0.518–0.979)	0.731 (0.531–1.005)	0.639 (0.459–0.889)	0.007	0.589 (0.401–0.863)
LDL-c (mmol/L)	1.75 [< 2.00]	2.19 [2.00-<2.34]	2.51 [2.34-<2.73]	3.11 [≥ 2.73]		
Model 1	Reference	1.264 (0.954–1.677)	1.420 (1.070–1.887)	1.398 (1.053–1.858)	0.014	1.217 (1.035–1.433)
Model 2	Reference	1.288 (0.939–1.768)	1.425 (1.034–1.967)	1.504 (1.086–2.086)	0.013	1.281 (1.064–1.544)
Triglyceride glucose index	0.75 [< 0.91]	1.03 [0.91-<1.13]	1.25 [1.13-<1.38]	1.57 [≥ 1.38]		
Model 1	Reference	0.928 (0.694–1.241)	1.520 (1.142–2.027)	2.315 (1.736–3.095)	< 0.001	2.470 (1.880–3.261)
Model 2	Reference	0.883 (0.636–1.223)	1.232 (0.892–1.701)	1.952 (1.398–2.731)	< 0.001	2.024 (1.492–2.761)
TG/HDL-c ratio	0.51 [< 0.60]	0.70 [0.60-<0.79]	0.91 [0.79-<1.07]	1.34 [≥ 1.07]		
Model 1	Reference	1.033 (0.774–1.380)	1.595 (1.200-2.124)	2.485 (1.865–3.321)	< 0.001	1.881 (1.495–2.396)
Model 2	Reference	1.089 (0.791–1.501)	1.424 (1.036–1.960)	2.127 (1.526–2.971)	< 0.001	1.513 (1.207–1.946)
TC/HDL-c ratio	2.29 [< 2.48]	2.63 [2.48-<2.78]	2.92 [2.78-<3.11]	3.45 [≥ 3.11]		
Model 1	Reference	1.461 (1.094–1.954)	1.903 (1.428–2.543)	2.603 (1.945–3.487)	< 0.001	1.828 (1.519–2.209)
Model 2	Reference	1.417 (1.027–1.957)	1.786 (1.292–2.473)	2.370 (1.700-3.312)	< 0.001	1.717 (1.381–2.141)

Model 1 was an unadjusted model. Model 2 was adjusted for maternal age, educational level, marital status, pre-pregnancy BMI, parity, mode of conception, gestational age at the time of blood collection in early pregnancy, and fertile season

*Tests for trend were assessed by modeling median values of quartiles of maternal lipid profile and lipid ratios as continuous variables

Abbreviations: BMI, body mass index; GDM, gestational diabetes mellitus; TC, total cholesterol; TG, triglyceride; LDL-c, low-density lipoprotein cholesterol; HDL-c, high-density lipoprotein cholesterol; ApoA1, Apolipoprotein AI; ApoB, Apolipoprotein B

### Association between lipid profiles and lipid ratios and GDM in middle pregnancy

Similar trends were observed only between triglyceride glucose index, HDL-c (mmol/L) and risk of GDM in the second trimester of pregnancy, and associations between the rest of the lipid profiles and indicators of pancreatic resistance and GDM have not been found (Table 3). As the 1-SD of Triglyceride glucose index, and HDL-c (mmol/L) increased, the OR (95% CIs) of GDM in model 2 (Table 3) were 1.884(1.076–3.358) and 0.537(0.336–0.852), respectively. In the fully corrected model, the OR (95% CI) for extreme interquartile comparisons of HDL-c, triglyceride glucose index, TG/HDL-c ratio, and TC/HDL-c ratio were 0.455(0.280–0.734), 2.318(1.278–4.262), respectively, 1.825(1.125–2.973) and 2.037(1.270–3.287) (*P* < 0.05 for trend). In addition, there was a lack of evidence for a non-linear association of TG, TC, ApoA1, ApoB, HDL-c, LDL-c, triglyceride glucose index, TG/HDL-c and TC/HDL-c with GDM in mid-pregnancy (non-linear *P* > 0.005, Figure S2).

**Table 3:** ORs (95% CIs) for GDM according to quartiles of maternal lipid profile and lipid ratios in middle pregnancy

Maternal lipid profile and lipid ratios	OR (95%CI) for GDM				*P* for trend*	Per 1-SD increment
	Quartile 1	Quartile 2	Quartile 3	Quartile 4		OR (95%CI)
Middle pregnancy						
TC (mmol/L)	5.03 [< 5.49]	5.83 [5.49-<6.14]	6.44 [6.14-<6.80]	7.24 [≥ 6.80]		
Model 1	Reference	0.613 (0.402–0.931)	0.843 (0.554–1.280)	0.844 (0.556–1.280)	0.762	0.928 (0.805–1.068)
Model 2	Reference	0.574 (0.359–0.913)	0.834 (0.524–1.327)	0.805 (0.503–1.286)	0.720	0.970 (0.826–1.139)
TG (mmol/L)	1.60 [< 1.85]	2.05 [1.85-<2.27]	2.55 [2.27-<2.86]	3.49 [≥ 2.86]		
Model 1	Reference	1.268 (0.831–1.936)	1.580 (1.041–2.407)	1.510 (0.994–2.302)	0.031	1.125 (0.976–1.308)
Model 2	Reference	1.231 (0.767–1.978)	1.532 (0.961–2.449)	1.301 (0.812–2.085)	0.111	1.031 (0.882–1.214)
ApoA1 (g/L)	1.47 [< 1.57]	1.68 [1.57-<1.76]	1.86 [1.76-<1.98]	2.15 [≥ 1.98]		
Model 1	Reference	1.230 (0.808–1.874)	0.954 (0.627–1.452)	0.744 (0.486–1.136)	0.099	0.727 (0.428–1.217)
Model 2	Reference	1.109 (0.696–1.769)	0.823 (0.514–1.317)	0.708 (0.439–1.140)	0.089	0.906 (0.495–1.632)
ApoB (g/L)	0.80 [< 0.90]	1.10 [0.90-<1.10]	1.20 [1.10-<1.20]	1.30 [≥ 1.20]		
Model 1	Reference	0.875 (0.612–1.252)	0.874 (0.546–1.394)	0.798 (0.505–1.257)	0.337	0.496 (0.261–0.928)
Model 2	Reference	0.800 (0.538–1.188)	0.851 (0.505–1.427)	0.832 (0.501–1.377)	0.495	0.589 (0.288–1.194)
HDL-c (mmol/L)	1.53 [< 1.68]	1.83 [1.68-<1.95]	2.06 [1.95-<2.18]	2.40 [≥ 2.18]		
Model 1	Reference	0.626 (0.412–0.947)	0.543 (0.356–0.825)	0.414 (0.268–0.635)	< 0.001	0.415 (0.274–0.620)
Model 2	Reference	0.590 (0.371–0.935)	0.511 (0.319–0.815)	0.455 (0.280–0.734)	< 0.001	0.537 (0.336–0.852)
LDL-c (mmol/L)	2.48 [< 2.82]	3.08 [2.82-<3.34]	3.58 [3.34-<3.84]	4.27 [≥ 3.84]		
Model 1	Reference	0.822(0.539–1.250)	0.998(0.657–1.517)	1.267 (0.836–1.925)	0.180	1.012 (0.851–1.203)
Model 2	Reference	0.865 (0.542–1.380)	1.106 (0.697–1.758)	1.295 (0.811–2.073)	0.154	1.076 (0.884–1.31)
Triglyceride glucose index	1.28 [< 1.44]	1.57 [1.44-<1.67]	1.80 [1.67-<1.93]	2.13 [≥ 1.93]		
Model 1	Reference	0.915 (0.547–1.529)	1.942 (1.162–3.268)	1.765 (1.063–2.950)	0.003	2.092 (1.300-3.436)
Model 2	Reference	0.976 (0.537–1.776)	1.616 (0.894–2.941)	2.318 (1.278–4.262)	0.008	1.884 (1.076–3.358)
TG/HDL-c ratio	0.71 [< 0.89]	1.04 [0.89-<1.19]	1.38 [1.19-<1.62]	2.01 [≥ 1.62]		
Model 1	Reference	1.795 (1.170–2.767)	2.174 (1.413–3.668)	2.146 (1.397–3.318)	< 0.001	1.215 (1.023–1.481)
Model 2	Reference	1.756 (1.091–2.842)	2.149 (1.327–3.504)	1.825 (1.125–2.973)	0.005	1.096 (0.917–1.345)
TC/HDL-c ratio	2.57 [< 2.81]	2.97 [2.81-<3.17]	3.35 [3.17-<3.58]	2.01 [≥ 3.58]		
Model 1	Reference	1.636 (1.068–2.517)	1.519 (0.995–2.328)	2.260 (1.475–3.484)	< 0.001	1.094 (0.949–1.336)
Model 2	Reference	1.300 (0.811–2.088)	1.322(0.827–2.117)	2.037 (1.270–3.287)	0.003	1.063 (0.915–1.244)

Model 1 was unadjusted. Model 2 was adjusted for maternal age, educational level, marital status, pre-pregnancy BMI, parity, mode of conception, gestational age at the time of blood collection in early pregnancy, and fertile season

*Tests for trend were assessed by modeling median values of quartiles of maternal lipid profile and lipid ratios as continuous variables

Abbreviations: BMI, body mass index; GDM, gestational diabetes mellitus; TC, total cholesterol; TG, triglyceride; LDL-c, low-density lipoprotein cholesterol; HDL-c, high-density lipoprotein cholesterol; ApoA1, Apolipoprotein AI; ApoB, Apolipoprotein B

### Association of Patterns of Change in lipid profiles and lipid ratios with GDM risk in early to middle pregnancy

In univariate analyses, stable high levels of TG, triglyceride glucose index, TG/HDL-c, and TC/HDL-c were positively associated with the risk of GDM compared with stable low levels, whereas the opposite was true for HDL-c, whose stable high levels were negatively associated with the risk of GDM (Table 4). Except for TG, the above correlations remained significant after adjustment for confounders, with OR(95% CI) of 2.369(1.438–3.940), 1.588(1.077–2.341), 1.921(1.309–2.829), and 0.599 (0.405–0.883) for GDM, respectively (Table 4).

**Table 4:** Multivariable-adjusted ORs (95% CIs) for the associations between the change patterns of maternal lipid profile and lipid ratios from early to middle pregnancy and GDM

Groups	Early pregnancy	Middle pregnancy	Model1 OR (95%CI)	Model2 OR (95%CI)
TC (mmol/L)				
	low	low	Reference	Reference
	low	high	1.067(0.663–1.710)	0.955(0.558–1.629)
	high	low	1.340(0.841–2.137)	1.140(0.680–1.909)
	high	high	1.266(0.888–1.806)	1.272(0.860–1.887)
TG (mmol/L)				
	low	low	Reference	Reference
	low	high	0.856(0.515–1.407)	0.912(0.516–1.591)
	high	low	1.193(0.725–1.956)	0.957(0.550–1.654)
	high	high	1.756(1.242–2.489)	1.443(0.977–2.131)
ApoA1(g/L)				
	low	low	Reference	Reference
	low	high	0.669(0.417–1.064)	0.734(0.435–1.228)
	high	low	1.464(0.917–2.349)	1.366(0.808–2.318)
	high	high	0.929(0.647–1.333)	0.820(0.544–1.232)
ApoB(g/L)				
	low	low	Reference	Reference
	low	high	0.741(0.398–1.347)	0.879(0.434–1.727)
	high	low	1.499(1.023–2.203)	1.579(1.028–2.432)
	high	high	1.153(0.789–1.685)	1.202(0.791–1.827)
HDL-c (mmol/L)				
	low	low	Reference	Reference
	low	high	0.563(0.340–0.925)	0.566(0.323–0.981)
	high	low	0.609(0.370–0.996)	0.667(0.381–1.159)
	high	high	0.536(0.378–0.758)	0.599(0.405–0.883)
LDL-c (mmol/L)				
	low	low	Reference	Reference
	low	high	0.920(0.579–1.456)	0.826(0.490–1.385)
	high	low	0.832(0.516–1.331)	0.782(0.459–1.320)
	high	high	1.379(0.965–1.973)	1.468(0.985–2.194)
Triglyceride glucose index				
	low	low	Reference	Reference
	low	high	1.607(0.858–3.006)	2.053(0.995–4.255)
	high	low	1.694(0.951–3.022)	1.366(0.695–2.686)
	high	high	2.589(1.682–4.015)	2.369(1.438–3.940)
TG/HDL-c ratio				
	low	low	Reference	Reference
	low	high	1.009(0.596–1.689)	1.289(0.720–2.291)
	high	low	1.423(0.851–2.376)	1.206(0.679–2.134)
	high	high	2.000(1.419–2.827)	1.588(1.077–2.341)
TC/HDL-c ratio				
	low	low	Reference	Reference
	low	high	0.685(0.403–1.142)	0.683(0.380–1.202)
	high	low	1.112(0.665–1.847)	1.049(0.594–1.844)
	high	high	1.984(1.404–2.813)	1.921(1.309–2.829)

Multivariable-adjusted ORs (95% CIs) for the associations between the change patterns of maternal lipid profile and lipid ratios from early to middle pregnancy and GDM

Model 1 was unadjusted. Model 2 was adjusted for maternal age, educational level, marital status, pre-pregnancy BMI, parity, mode of conception, gestational age at the time of blood collection in early pregnancy, and fertile season

High means levels above the median; while low means levels below the median

Abbreviations: BMI, body mass index; GDM, gestational diabetes mellitus; TC, total cholesterol; TG, triglyceride; LDL-c, low-density lipoprotein cholesterol; HDL-c, high-density lipoprotein cholesterol; ApoA1, Apolipoprotein AI; ApoB, Apolipoprotein B

### The combined effect of lipid profiles and lipid ratios and their corresponding patterns of change at different times and pre-pregnancy BMI on GDM risk

Elevated BMI is thought to be caused by poor lipid levels with high concentrations of TG, TC, ApoA1, ApoB, LDL-c and low concentrations of HDL-c [21]. We observed a positive association between pre-pregnancy BMI and GDM (Figure S3). Therefore, we investigated the combined effect of lipid profiles and lipid ratios and their corresponding patterns of change in early and mid-pregnancy and pre-pregnancy BMI on the risk of GDM.

**Fig. 2:**
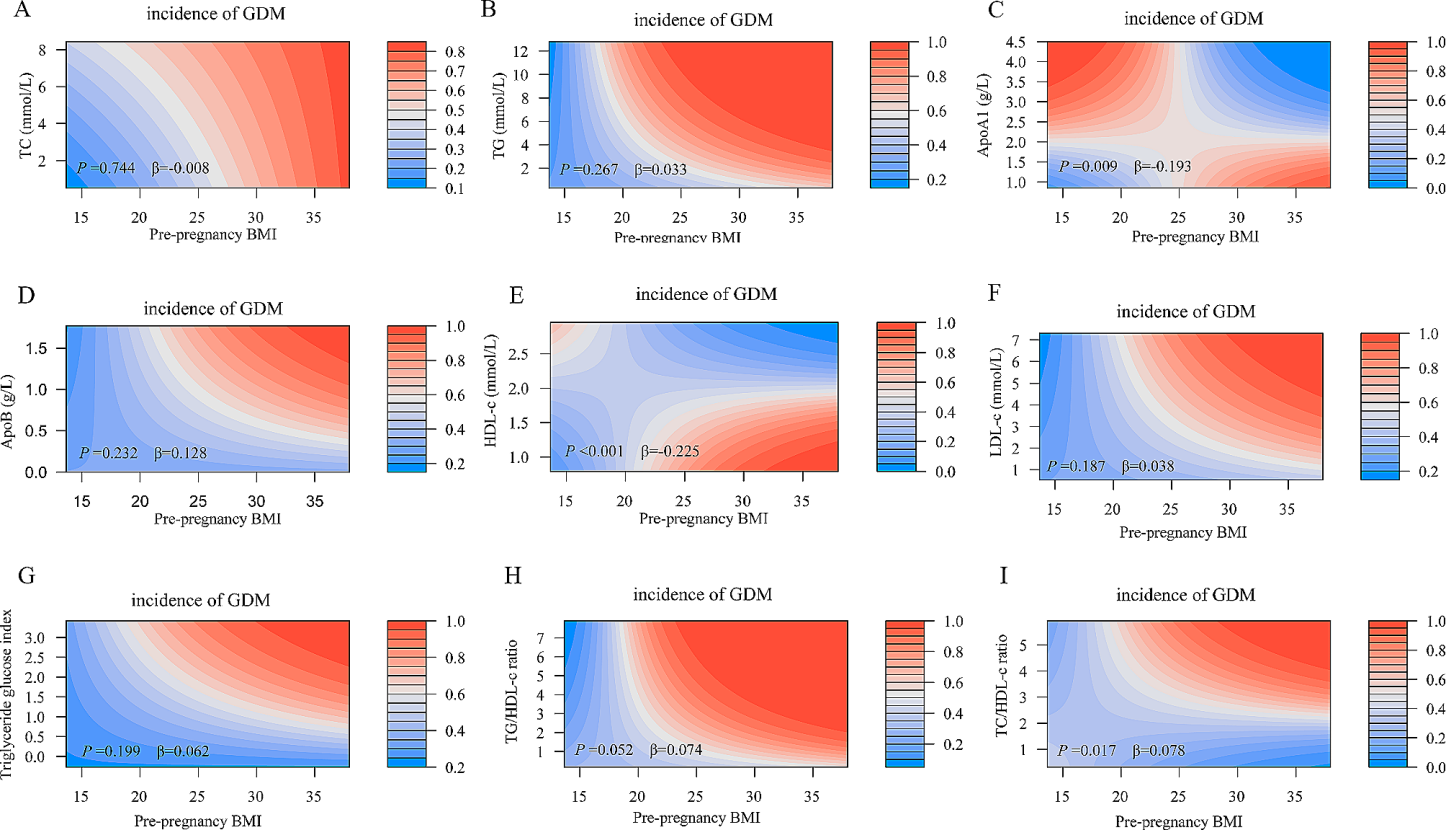
Combined effects of maternal pre-pregnancy BMI and maternal lipid profile and lipid ratios in early pregnancy on the incidence of GDM. Heat map for the correlation of incidence of GDM (red represents increased risks of GDM, blue represents decreased risks of GDM) according to the interaction of pre-pregnancy BMI and (**A**) TC, (**B**) TG, (**C**) ApoA1, (**D**) ApoB, (**E**) HDL-c, (**F**) LDL-c, (**G**) Triglyceride glucose index, (**H**) TG/HDL-c ratio, or (**I**) TC/HDL-c ratio. Analyses were adjusted for maternal age, educational level, marital status, pre-pregnancy BMI, parity, mode of conception, gestational age at the time of blood collection in early pregnancy, and fertile season

**Fig. 3:**
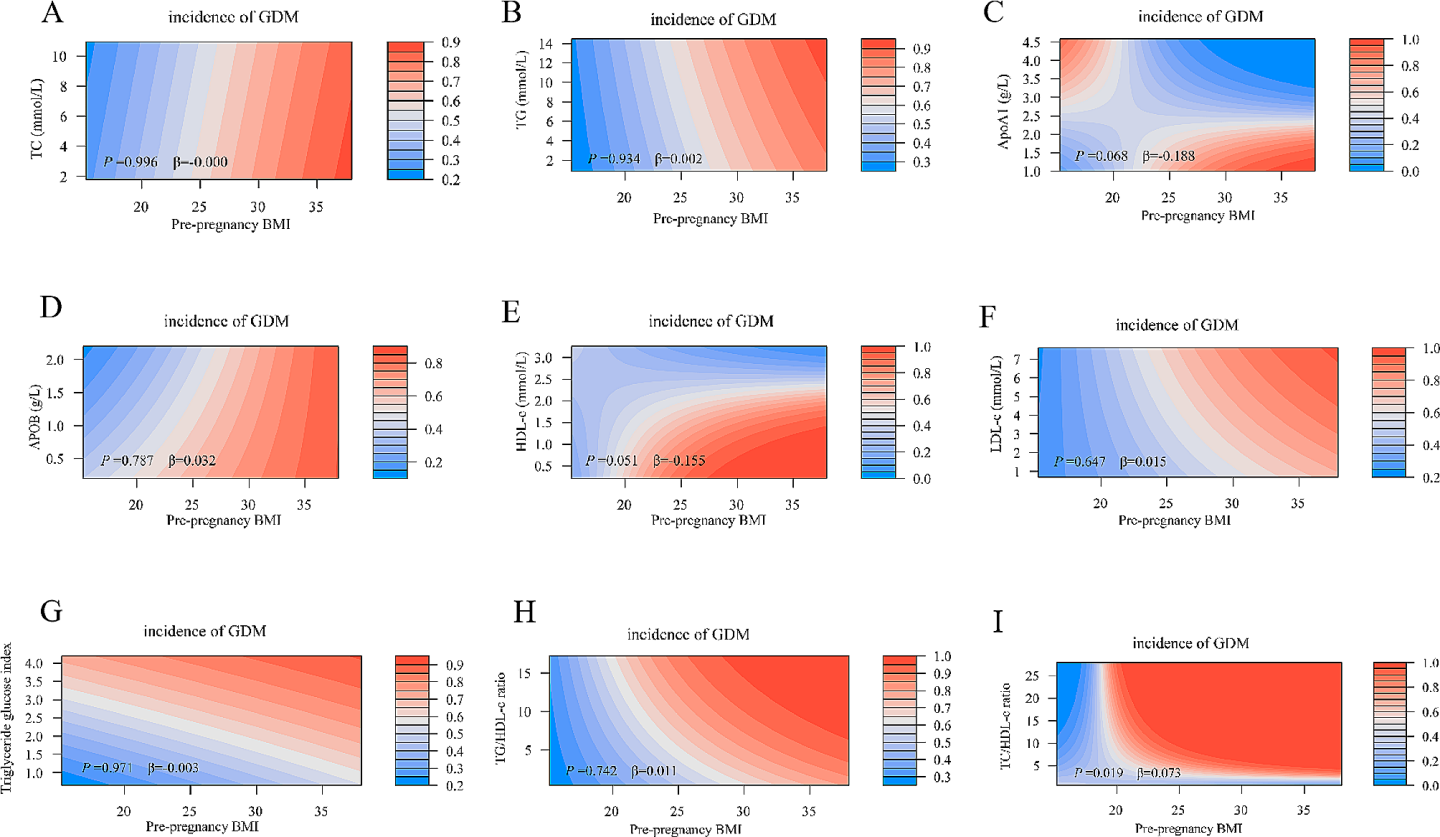
Combined effects of maternal pre-pregnancy BMI and maternal lipid profile and lipid ratios in middle pregnancy on the incidence of GDM. Heat map for the correlation of incidence of GDM (red represents increased risks of GDM, blue represents decreased risks of GDM) according to the interaction of pre-pregnancy BMI and (**A**) TC, (**B**) TG, (**C**) ApoA1, (**D**) ApoB, (**E**) HDL-c, (**F**) LDL-c, (**G**) Triglyceride glucose index, (**H**) TG/HDL-c ratio, or (**I**) TC/HDL-c ratio. Analyses were adjusted for maternal age, educational level, marital status, pre-pregnancy BMI, parity, mode of conception, gestational age at the time of blood collection in early pregnancy, and fertile season

Figures 2 and 3 show a heat map of the combined association of pre-pregnancy BMI (x-axis) (ranging from 14.95 kg/m2 to 37.97 kg/m2) and early/mid-pregnancy TC, TG, ApoA1, ApoB, HDL-c, LDL-c, triacylglycerol glucose index, TG/HDL-c, and TC/HDL-c (y-axis) with the incidence (%) of GDM (z-axis; red represents a higher incidence and blue a lower incidence). (z-axis; red represents higher incidence, blue represents lower incidence). A significant difference in the incidence of GDM was found between pre-pregnancy BMI and the combination of ApoA1, HDL-c, and TC/HDL-c based on the interaction terms of pre-pregnancy BMI and early gestation TC, TG, ApoA1, ApoB, HDL-c, LDL-c, triglyceride glucose index, TG/HDL-c, and TC/HDL-c (*P* for interaction < 0.05; Fig. 2). The interaction effect showed that an increase in TC/HDL-c and pre-pregnancy body mass index in early pregnancy was associated with a higher risk of GDM. In contrast, a decrease in ApoA1 and HDL-c in early pregnancy and an increase in prepregnancy body mass index were associated with a higher risk of GDM. A similar effect was observed in the combination of pre-pregnancy BMI and mid-pregnancy TC/HDL-c levels (*P* for interaction = 0.019) (Fig. 3). Testing of the interaction term between pre-pregnancy BMI and the pattern of change in lipid profile and lipid ratios from early to mid-pregnancy (Table S1) revealed that only the pattern of change in triglyceride glucose index from early to mid-pregnancy with pre-pregnancy BMI also had a combined effect on GDM (*P* < 0.05).

### Association between lipid profiles and lipid ratios and plasma glucose values in the OGTT

In early pregnancy, the levels of triglyceride glucose index, TG/HDL-c ratio, and TC/HDL-c ratio are positively correlated with fasting, 1-hour, and 2-hour OGTT glucose levels. The increase of TG and ApoA1 are positively correlated with 1-hour and 2-hour OGTT glucose levels. The increase of HDL-c is negatively correlated with fasting and 1-hour OGTT glucose levels. In mid-pregnancy, the levels of triglyceride glucose index and TG/HDL-c ratio increase, and HDL-c decreases are positively correlated with fasting, 1-hour, and 2-hour OGTT glucose levels. The increase of TG and ApoA1 is positively correlated with 1-hour and 2-hour OGTT glucose levels. Observing the changes of blood lipid spectrum and lipid ratios from early to mid-pregnancy, it is found that compared with the stable low level, the stable high level of triglyceride glucose index, TG/HDL-c ratio, and TC/HDL-c ratio are positively correlated with fasting, 1-hour, and 2-hour OGTT glucose levels (Table S2).

## Discussion

In this prospective study exploring the correlation of lipid profiles and lipid ratios with subsequent GDM risk in early to mid-pregnancy, we found that TG, ApoA1, ApoB, LDL-c, triglyceride glucose index, TG/HDL-c, and TC/HDL-c were positively correlated with the risk of GDM in early gestation, whereas HDL-c was negatively associated with GDM risk. In addition, the associations of HDL-c, triglyceride glucose index, TG/HDL-c ratio, and TC/HDL-c ratio with GDM persisted in mid-gestation, and their consistently high levels from early to mid-gestation were positively associated with the risk of GDM, except for HDL-c, which was negatively associated with the risk of GDM. This provides a possible biological mechanism for the early prevention and management of GDM.

In early pregnancy, TG, ApoA1, ApoB, LDL-c, triglyceride glycaemic index, TG/HDL-c and TC/HDL-c were positively correlated with the risk of GDM, whereas HDL-c was negatively correlated with the risk of GDM, which is consistent with previous reports [22–27]. However, in a study conducted in China [28], women with GDM had higher concentrations of TG and lower levels of HDL-c compared with controls, and there was a statistically significant difference, whereas other TC, LDL-c, APOA1 and APOB did not show statistically significant differences between the two groups. In addition, a meta-analysis [29] found that women with GDM had higher TG and lower HDL-c in early and mid-pregnancy compared to women with healthy pregnancies.

In studies exploring the relationship between lipid profile and GDM [30], TG is the most critical. Most of the included studies reported that higher TG levels in women with GDM occurred in early pregnancy and persisted throughout pregnancy, which is relatively similar to the results in early pregnancy in our study, but there is a difference in that in the mid-pregnancy the association between TG and GDM did not appear statistically different, but instead higher HDL-c and its pattern of persistent high level changes in early and mid-pregnancy were associated with a reduced risk of GDM significantly associated with GDM, which is partly supported by another study [8]. This is partly supported by other studies [19, 31, 32] that showed no significant association between lipids and GDM in early and mid-pregnancy. Differences in the results of these studies may be due, at least in part, to heterogeneity in study design and study methodology, such as differences in gestational age, fasting status and diagnostic criteria for GDM at the time of blood collection, inconsistent or inadequate control of confounders, and differences in population characteristics [19]. More longitudinal studies with large sample sizes are necessary to determine the role of lipid metabolism in the development of GDM in populations.

The triacylglycerol glucose index [24], a comprehensive biochemical indicator reflecting the combined effects of lipids and glucose, the TG/HDL-c ratio [33], which combines TG and HDL-c, and the TC/HDL-c ratio [34] were all associated with the risk of GDM, which may be related to insulin resistance, which is consistent with our findings. Although all three indices seem to reflect insulin resistance in pregnant women, further studies are essential to fully explore their potential for early prediction of GDM. Meanwhile, the association of these three lipid ratios with GDM is more commonly seen in cross-sectional studies focusing on early pregnancy, and no studies have been seen to take into account the physiological changes of pregnancy in women.

The associations of changes in lipid profile and associated lipid ratios with GDM in early to mid-gestation have been less frequently studied. In our study, the associations of HDL-c, triglyceride glucose index, TG/HDL-c ratio, and TC/HDL-c ratio with GDM persisted in mid-gestation, and their consistently high levels from early to mid-gestation were positively associated with the risk of GDM, except for HDL-c, which was negatively associated with the risk of GDM, suggesting that the pattern of changes in lipid profiles and lipid ratios may be as meaningful as their levels in relation to the development of GDM in mid-gestation. Of note, lipid profiles and lipid ratios in early and mid-pregnancy were mostly positively associated with glucose levels at the 3 different time points in the OGTT, except for HDL-c, which was associated with the opposite risk, regardless of whether or not the relationship was statistically different. Given the limited data on the pattern of changes in lipid profile and lipid ratio during pregnancy, our findings require further confirmation, especially in the Chinese population.

Although the mechanisms underlying the relationship between lipid profiles, lipid ratios and GDM remain to be elucidated, our findings are biologically plausible. During pregnancy, elevated oestrogen levels and insulin resistance promote lipid synthesis in the liver [29], while at the same time the mother uses lipids as a source of energy to promote fetal growth and development to conserve glucose, both suggesting that the physiological changes in a woman’s body during pregnancy do not prioritise glucose metabolism, but rather lipid metabolism. In early pregnancy, lipids increase, thereby raising blood levels of free fatty acids, which may impair insulin sensitivity and create a vicious circle between high lipid levels and IR, leading to impaired glucose tolerance and the development of diabetes mellitus [35]. In addition. Reduced insulin secretion, reduced insulin sensitivity and reduced AMP-activated protein kinase activity are all possible effects of low HDL-C levels on glucose homeostasis [36].

The strengths of this study include a prospective design, a larger sample size and a well-characterised study population with more comprehensive and standardised medical records. In addition, venous blood was drawn in the fasting state to better reflect lipid metabolic status. However, several limitations of this study should be acknowledged. First, although all existing confounding factors have been corrected, due to the limitations of the study, some confounding factors were not collected, such as history of GDM in previous pregnancies, history of lipid profile disorders, lifestyle factors, dietary factors, and other factors. We found extremely low rates of smoking and alcohol consumption among pregnant women, who were more inclined to provide proactive answers considering that most of them were accompanied by family members during antenatal visits. This social desirability factor could have led to inaccuracies in these two variables, and therefore, we did not include them in our analyses. Second, we did not have data on insulin resistance or direct measurement of insulin homeostasis to explore the potential mechanisms. Finally, in our nested case-control study, due to time constraints, the follow-up time is not long enough, and the sample size is also small. In future research, the sample size should be expanded. research, the sample size should be expanded and the follow-up time should be extended to obtain more credible conclusions.

## Conclusions

Overall, early and mid-pregnancy triglyceride glucose index, TG/HDL-c ratio, and TC/HDL-c ratio and their stable high levels are associated with a higher risk of GDM, and the opposite is true for HDL-c. In addition, early and mid-pregnancy screening for lipid profiles and lipid ratios and pre-pregnancy weight status assessment are essential for filtering mothers prone to GDM. More studies focusing on the impact of lipid profiles and lipid ratios during pregnancy are necessary considering maternal health.

### Electronic supplementary material

Below is the link to the electronic supplementary material.

Supplementary Material 1

## Data Availability

The datasets generated and/or analysed during the current study are not publicly available due to the principle of confidentiality of funding but are available from the corresponding author on reasonable request.
