# Supplementary material for: Association between maternal lipid profiles and lipid ratios in early to middle pregnancy as well as their dynamic changes and gestational diabetes mellitus
Source: BMC Pregnancy Childbirth. 2024 Jul 29;24:510. doi: 10.1186/s12884-024-06692-9 (PMC11285337; doi:10.1186/s12884-024-06692-9)
Supplement: Supplementary file 1 — Supplementary Material 1 [file 12884_2024_6692_MOESM1_ESM.docx]

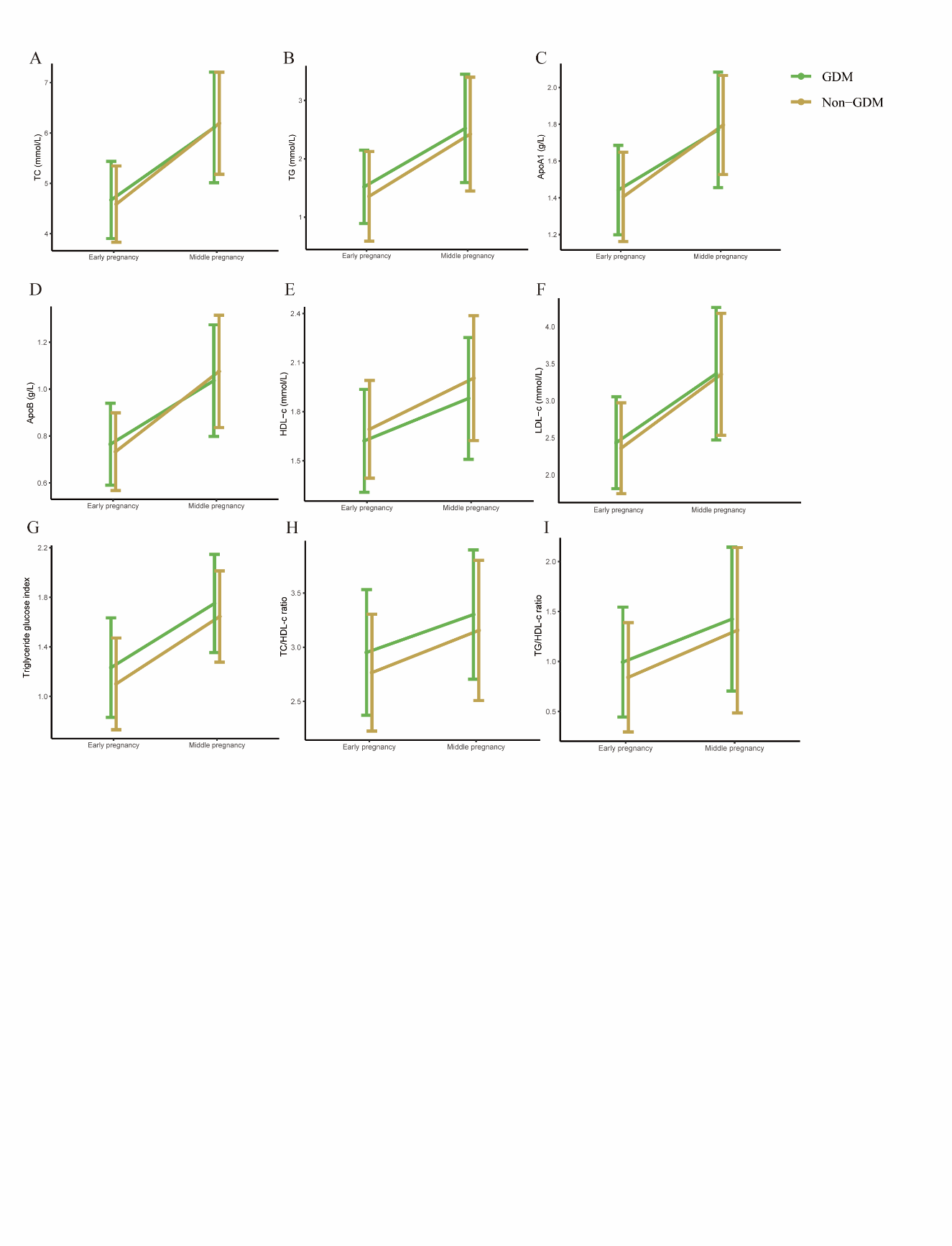


Figure S1 Mean (95% CI) of maternal lipid profile and markers of insulin resistance from early to middle pregnancy among women with and without GDM. Abbreviations: GDM, gestational diabetes mellitus; TC, total cholesterol; TG, triglyceride; LDL-c, low-density lipoprotein cholesterol; HDL-c, high-density lipoprotein cholesterol; ApoA1, Apolipoprotein AI; ApoB, Apolipoprotein B.


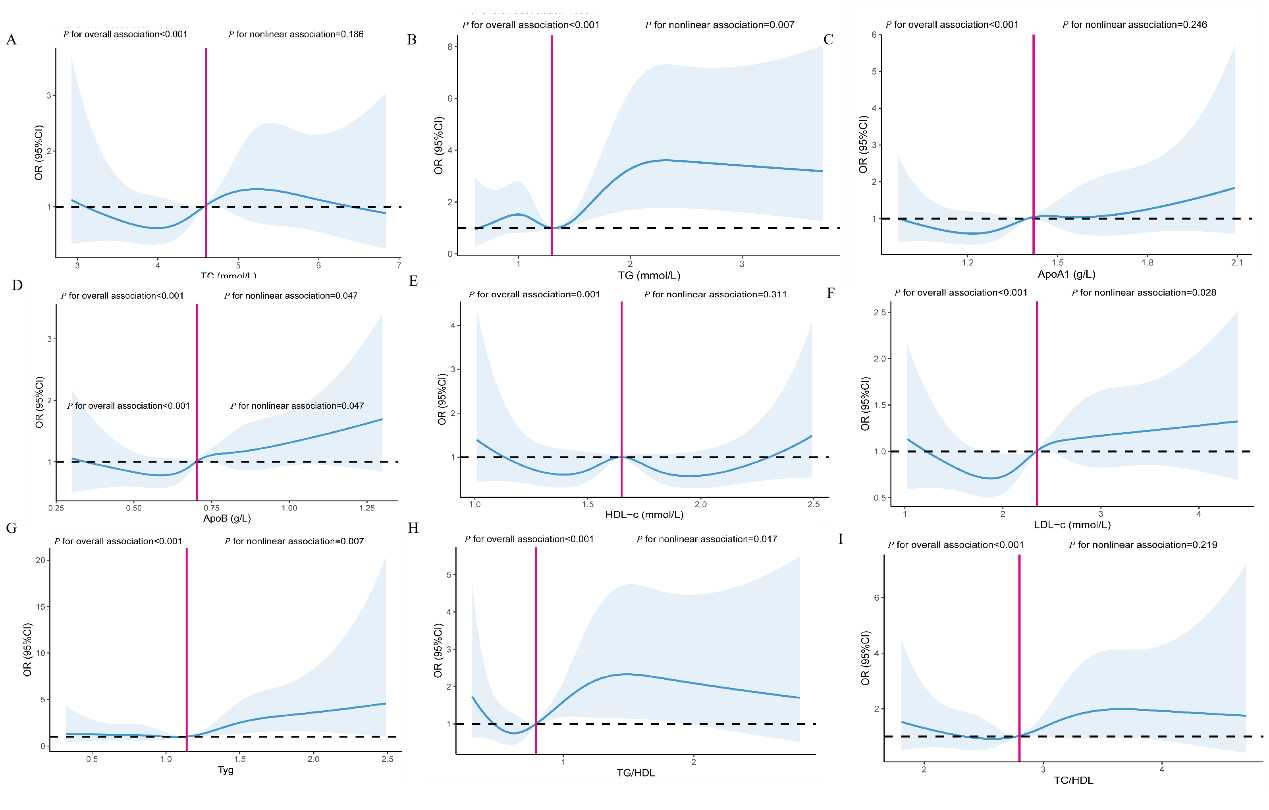


Figure S2 Restricted cubic splines-based modeling for the associations of maternal lipid profile and markers of insulin resistance in middle pregnancy with GDM. The model was adjusted for maternal age, educational level, marital status, pre-pregnancy BMI, parity, mode of conception, gestational age at the time of blood collection in early pregnancy, and fertile season. Panels A-I were respectively TC, TG, ApoA1, ApoB, HDL-c, LDL-c, Tyg, TG/HDL and TC/HDL. Abbreviations: BMI, body mass index; GDM, gestational diabetes mellitus; TC, total cholesterol; TG, triglyceride; LDL-c, low-density lipoprotein cholesterol; HDL-c, high-density lipoprotein cholesterol; ApoA1, Apolipoprotein AI; ApoB, Apolipoprotein B.


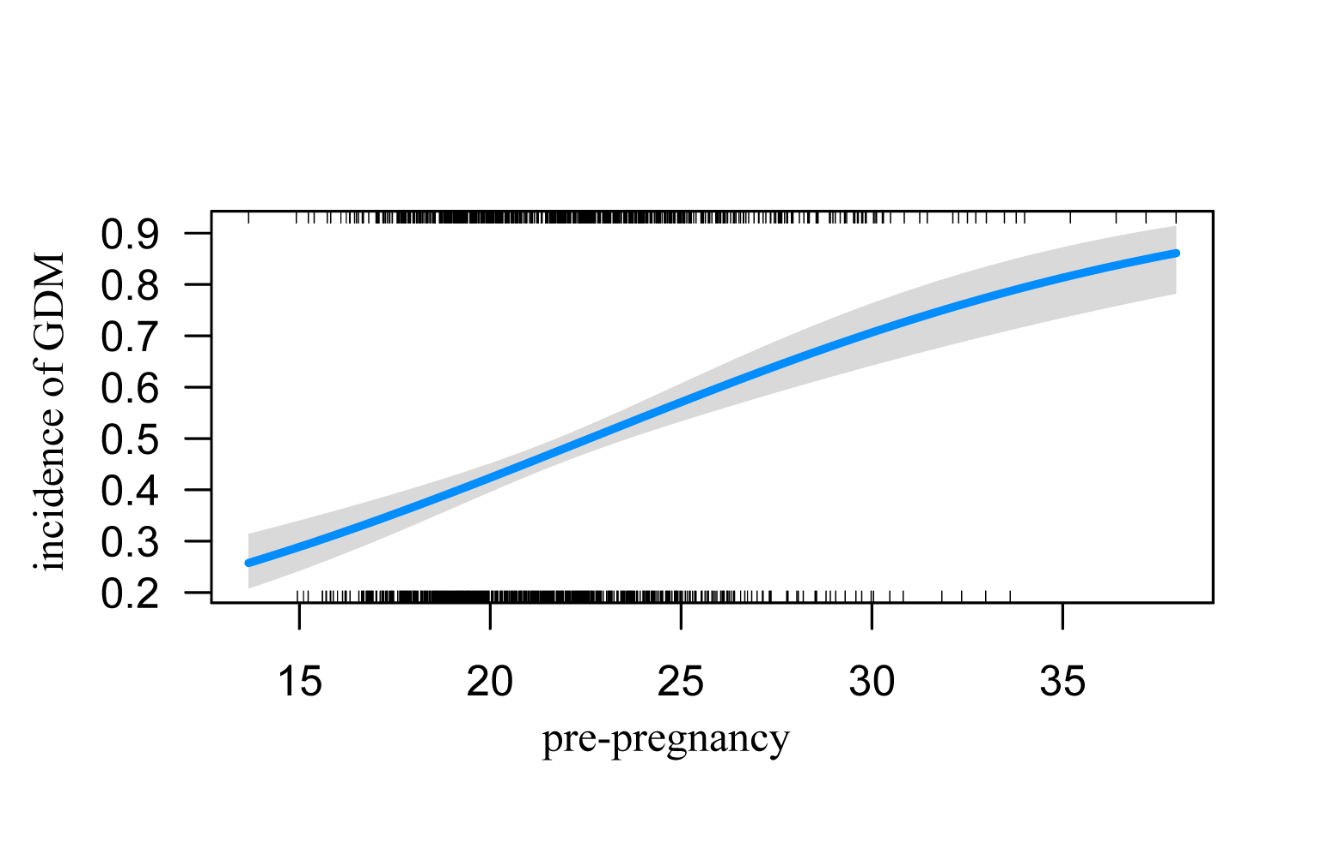


Figure S3 The association between pre-pregnancy and GDM. The solid blue line represents the smooth curve fit between variables. Grey bands represent the 95% confidence interval from the fit.

**Table S1 Combined effects of maternal pre-pregnancy BMI and the change patterns of maternal lipid profile and lipid ratios on the incidence of GDM.**

| **Groups** | **Early pregnancy** | **Middle pregnancy** | **Model**  **OR (95%CI)** | **P for interaction** |
| --- | --- | --- | --- | --- |
| TC (mmol/L) |  |  |  | 0.946 |
|  | low | low | Reference |  |
|  | low | high | 1.060(0.854-1.328) |  |
|  | high | low | 1.025(0.872-1.208) |  |
|  | high | high | 1.030(0.904-1.173) |  |
| TG (mmol/L) |  |  |  | 0.069 |
|  | low | low | Reference |  |
|  | low | high | 0.953(0.765- 1.190) |  |
|  | high | low | 1.296(1.053-1.640) |  |
|  | high | high | 1.042(0.919-1.183) |  |
| ApoA1(g/L) |  |  |  | 0.411 |
|  | low | low | Reference |  |
|  | low | high | 0.965(0.817-1.145) |  |
|  | high | low | 1.034(0.872-1.237) |  |
|  | high | high | 0.910(0.796- 1.039) |  |
| ApoB(g/L) |  |  |  | 0.732 |
|  | low | low | Reference |  |
|  | low | high | 0.925(0.702-1.182) |  |
|  | high | low | 1.029(0.888-1.197) |  |
|  | high | high | 1.054(0.921-1.212) |  |
| HDL-c (mmol/L) |  |  |  | 0.642 |
|  | low | low | Reference |  |
|  | low | high | 0.951(0.785- 1.158) |  |
|  | high | low | 0.947(0.804- 1.119) |  |
|  | high | high | 0.917(0.802- 1.048) |  |
| LDL-c (mmol/L) |  |  |  | 0.946 |
|  | low | low | Reference |  |
|  | low | high | 0.988(0.805- 1.214) |  |
|  | high | low | 1.014(0.859- 1.201) |  |
|  | high | high | 1.037(0.904- 1.190) |  |
| Triglyceride glucose index |  |  |  | 0.022 |
|  | low | low | Reference |  |
|  | low | high | 0.900(0.682-1.147) |  |
|  | high | low | 1.343(1.061-1.765) |  |
|  | high | high | 1.115(0.949-1.316) |  |
| TG/HDL-c ratio |  |  |  | 0.238 |
|  | low | low | Reference |  |
|  | low | high | 1.129(0.912-1.432) |  |
|  | high | low | 1.223(0.997-1.533) |  |
|  | high | high | 1.052(0.926-1.196) |  |
| TC/HDL-c ratio |  |  |  | 0.707 |
|  | low | low | Reference |  |
|  | low | high | 1.060(0.860-1.319) |  |
|  | high | low | 1.117(0.898-1.408) |  |
|  | high | high | 1.062(0.936- 1.204) |  |

Multivariable-adjusted ORs (95% CIs) for the associations between the change patterns of maternal lipid profile and lipid ratios from early to middle pregnancy and GDM.

The model was adjusted for maternal age, educational level, marital status, pre-pregnancy BMI, parity, mode of conception, gestational age at the time of blood collection in early pregnancy, and fertile season. High means the levels above the median; while low means the levels below the median.

**Table S2 Multivariable-adjusted regression coefficients for the association between maternal lipid profile and lipid ratios and plasma glucose values in 75-g OGTT**

| **Maternal lipid profile and lipid ratios** | **OGTT-fasting** | | **OGTT-1 hour** | | **OGTT-2 hour** | |
| --- | --- | --- | --- | --- | --- | --- |
|  | **β (95%CI)** | ***P* value** | **β (95%CI)** | ***P* value** | **β (95%CI)** | ***P* value** |
| Early pregnancy |  |  |  |  |  |  |
| TC (mmol/L) | -0.001  (-0.028-0.0269) | 0.964 | 0.051  (-0.073-0.174) | 0.420 | 0.062  (-0.044-0.168) | 0.249 |
| TG (mmol/L) | 0.0645  (0.031-0.0980) | <0.001 | 0.139  (-0.010-0.288) | 0.069 | 0.200  (0.071-0.328) | 0.002 |
| ApoA1(g/L) | -0.0200  (-0.107-0.067) | 0.654 | 0.445  (0.059-0.832) | 0.024 | 0.513  (0.181-0.846) | 0.003 |
| ApoB(g/L) | 0.123  (-0.003-0.250) | 0.056 | 0.378  (-0.186-0.941) | 0.189 | 0.588  (0.103-1.073) | 0.018 |
| HDL-c (mmol/L) | -0.169  (-0.239--0.098) | <0.001 | -0.330  (-0.646--0.014) | 0.041 | -0.172  (-0.445-0.100) | 0.215 |
| LDL-c (mmol/L) | 0.034  (-0.001-0.069) | 0.055 | 0.107  (-0.048-0.261) | 0.177 | 0.085  (-0.048-0.218) | 0.211 |
| Triglyceride glucose index | 0.205  (0.148-0.263) | <0.001 | 0.416  (0.158-0.674) | 0.002 | 0.486  (0.257-0.715) | <0.001 |
| TG/HDL-c ratio | 0.108  (0.064-0.152) | <0.001 | 0.231  (0.036-0.427) | 0.021 | 0.255  (0.086-0.424) | 0.003 |
| TC/HDL-c ratio | 0.095  (0.056-0.135) | <0.001 | 0.268  (0.092-0.444) | 0.003 | 0.202  (0.050-0.355) | 0.010 |
| Middle pregnancy |  |  |  |  |  |  |
| TC (mmol/L) | -0.033  (-0.063 -0.0023) | 0.035 | -0.095  (-0.227-0.038) | 0.162 | -0.021  (-0.137-0.094) | 0.720 |
| TG (mmol/L) | 0.049  (0.019- 0.079) | 0.001 | 0.073  (-0.057- 0.203) | 0.270 | 0.171  (0.060- 0.282) | 0.003 |
| ApoA1(g/L) | -0.089  (-0.199- 0.021) | 0.115 | -0.177  (-0.661- 0.307) | 0.484 | 0.004  (-0.418-0.425) | 0.986 |
| ApoB(g/L) | -0.097  (-0.229- 0.035) | 0.152 | -0.363  (-0.936- 0.209) | 0.214 | -0.173  (-0.675-0.328) | 0.499 |
| HDL-c (mmol/L) | -0.227  (-0.311- -0.143) | <0.001 | -0.591  (-0.964--0.218) | 0.002 | -0.485  (-0.810--0.160) | 0.004 |
| LDL-c (mmol/L) | -0.015  (-0.052- 0.022) | 0.436 | -0.039  (-0.201- 0.124) | 0.641 | -0.002  (-0.144-0.141) | 0.982 |
| Triglyceride glucose index | 0.294  (0.196-0.393) | <0.001 | 0.605  (0.167- 1.043) | 0.007 | 0.710  (0.331-1.089) | 0.040 |
| TG/HDL-c ratio | 0.085  (0.055- 0.116) | <0.001 | 0.131  (-0.005- 0.268) | 0.060 | 0.182  (0.064- 0.299) | 0.003 |
| TC/HDL-c ratio | 0.034  (0.008- 0.060) | 0.011 | 0.068  (-0.046- 0.182) | 0.242 | 0.063  (-0.036-0.162) | 0.211 |
| Changes from early to middle pregnancy |  |  |  |  |  |  |
| TC (mmol/L) |  |  |  |  |  |  |
| Low, low | Reference |  | Reference |  | Reference |  |
| Low, high | -0.026  (-0.124- 0.073) | 0.611 | -0.376  (-0.807-0.0558) | 0.088 | 0.023  (-0.354-0.400) | 0.906 |
| High, low | -0.011  (-0.111- 0.089) | 0.824 | -0.251  (-0.686- 0.184) | 0.259 | 0.018  (-0.361-0.397) | 0.925 |
| High, high | -0.012  (-0.087- 0.062) | 0.747 | -0.051  (-0.378- 0.275) | 0.758 | 0.069  (-0.216-0.354) | 0.636 |
| TG (mmol/L) |  |  |  |  |  |  |
| Low, low | Reference |  | Reference |  | Reference |  |
| Low, high | 0.074  ( -0.028- 0.176) | 0.154 | 0.190  (-0.257- 0.637) | 0.406 | 0.240  (-0.148-0.627) | 0.226 |
| High, low | 0.050  (-0.053- 0.152) | 0.342 | 0.066  (-0.392- 0.524) | 0.777 | -0.018  (-0.410-0.374) | 0.930 |
| High, high | 0.046  (-0.029- 0.121) | 0.233 | 0.216  (-0.113-0.545) | 0.199 | 0.426  (0.141-0.711) | 0.004 |
| ApoA1(g/L) |  |  |  |  |  |  |
| Low, low | Reference |  | Reference |  | Reference |  |
| Low, high | -0.067  (-0.164- 0.029) | 0.173 | -0.301  (-0.727-0.125) | 0.167 | -0.126  (-0.500-0.247) | 0.507 |
| High, low | -0.011  (-0.111- 0.088) | 0.820 | -0.013  (-0.448-0.422) | 0.953 | 0.091  (-0.290-0.473) | 0.640 |
| High, high | -0.065  (-0.142- 0.012) | 0.100 | -0.039  (-0.380-0.301) | 0.821 | 0.106  (-0.193-0.404) | 0.488 |
| ApoB(g/L) |  |  |  |  |  |  |
| Low, low | Reference |  | Reference |  | Reference |  |
| Low, high | -0.003  (-0.124- 0.119) | 0.966 | -0.043  (-0.583-0.497) | 0.876 | 0.280  (-0.184-0.743) | 0.237 |
| High, low | -0.058  (-0.024- 0.140) | 0.165 | 0.154  (-0.205-0.513) | 0.402 | 0.376  (0.065-0.687) | 0.018 |
| High, high | -0.012  (-0.091- 0.068) | 0.777 | 0.050  (-0.302-0.402) | 0.780 | 0.044  (-0.263-0.350) | 0.781 |
| HDL-c (mmol/L) |  |  |  |  |  |  |
| Low, low | Reference |  | Reference |  | Reference |  |
| Low, high | -0.098  (-0.198-0.002) | 0.055 | -0.309  (-0.758-0.140) | 0.177 | -0.303  (-0.700-0.089) | 0.130 |
| High, low | -0.131  (-0.235- -0.028) | 0.013 | -0.147  (-0.602-0.309) | 0.528 | -0.071  (-0.46928034 0.327) | 0.726 |
| High, high | -0.151  (-0.225--0.076) | <0.001 | -0.243  (-0.574-0.088) | 0.151 | -0.276  (-0.565-0.014) | 0.062 |
| LDL-c (mmol/L) |  |  |  |  |  |  |
| Low, low | Reference |  | Reference |  | Reference |  |
| Low, high | 0.041  (-0.081- 0.109) | 0.771 | -0.214  (-0.628- 0.201) | 0.313 | -0.070  (-0.433-0.294) | 0.708 |
| High, low | 0.030  (-0.071- 0.132) | 0.556 | -0.019  (-0.463- 0.424) | 0.932 | 0.111  (-0.275-0.500) | 0.574 |
| High, high | 0.023  (-0.053- 0.099) | 0.550 | 0.148  (-0.185- 0.480) | 0.385 | 0.131  (-0.161-0.423) | 0.380 |
| Triglyceride glucose index |  |  |  |  |  |  |
| Low, low | Reference |  | Reference |  | Reference |  |
| Low, high | 0.128  (0.003- 0.252) | 0.045 | 0.013  (-0.544- 0.569) | 0.965 | 0.041  (-0.441-0.523) | 0.867 |
| High, low | 0.032  (-0.085-0.148) | 0.593 | -0.167  (-0.689- 0.355) | 0.531 | 0.030  (-0.424-0.483) | 0.898 |
| High, high | 0.213  (0.123-0.303) | <0.001 | 0.468  (0.069-0.866) | 0.022 | 0.575  (0.227-0.923) | 0.001 |
| TG/HDL-c ratio |  |  |  |  |  |  |
| Low, low | Reference |  | Reference |  | Reference |  |
| Low, high | -0.011  (-0.116-0.094) | 0.834 | 0.203  (-0.263- 0.667) | 0.394 | 0.165  (-0.239-0.569) | 0.424 |
| High, low | 0.095  (-0.010-0.201) | 0.077 | 0.261  (-0.214- 0.735) | 0.282 | 0.035  (-0.375- 0.444) | 0.868 |
| High, high | 0.096  （0.021- 0.172) | 0.013 | 0.333  (0.003- 0.664) | 0.048 | 0.437  (0.140-0.714) | 0.004 |
| TC/HDL-c ratio |  |  |  |  |  |  |
| Low, low | Reference |  | Reference |  | Reference |  |
| Low, high | 0.003  (-0.097- 0.104) | 0.949 | 0.009  (-0.436- 0.455) | 0.968 | 0.149  (-0.239-0.537) | 0.453 |
| High, low | 0.103  (-0.004-0.209) | 0.589 | 0.280  (-0.189- 0.748) | 0.243 | 0.024  (-0.387-0.435) | 0.908 |
| High, high | 0.136  (0.062-0.210) | <0.001 | 0.362  (0.036- 0.688) | 0.030 | 0.305  (0.021-0.589) | 0.036 |

High means levels above median, while low means levels below median.

Abbreviations: BMI, body mass index; TC, total cholesterol; TG, triglyceride; LDL-c, low-density lipoprotein cholesterol; HDL-c, high-density lipoprotein cholesterol; ApoA1, Apolipoprotein AI; ApoB, Apolipoprotein B; OGTT, oral glucose tolerance test.

The β coefficients are derived from multiple linear regression models.

Models were adjusted for maternal age, educational level, marital status, pre-pregnancy BMI, parity, mode of conception, gestational age at the time of blood collection in early pregnancy, and fertile season.
